# Context-specific, evidence-based planning for scale-up of family planning services to increase progress to MDG 5: health systems research

**DOI:** 10.1186/1742-4755-9-27

**Published:** 2012-11-12

**Authors:** Abbey Byrne, Alison Morgan, Eliana Jimenez Soto, Zoe Dettrick

**Affiliations:** 1 Nossal Institute for Global Health, University of Melbourne, Level 4, Alan Gilbert Building 161 Barry Street, Carlton, VIC, 3010, Australia; 2 School of Population Health, University of Queensland, 4th Floor, Public Health Building, Herston Road, Herston, QLD, 4006, Australia

**Keywords:** Family planning, Maternal mortality, Health systems research, Health planning, Evidence based planning, Indonesia, Philippines, Nepal

## Abstract

**Background:**

Unmet need for family planning is responsible for 7.4 million disability-adjusted life years and 30% of the maternity-related disease burden. An estimated 35% of births are unintended and some 200 million couples state a desire to delay pregnancy or cease fertility but are not using contraception. Unmet need is higher among the poorest, lesser educated, rural residents and women under 19 years. The barriers to, and successful strategies for, satisfying all demand for modern contraceptives are heavily influenced by context. Successfully overcoming this to increase the uptake of family planning is estimated to reduce the risk of maternal death by up to 58% as well as contribute to poverty reduction, women’s empowerment and educational, social and economic participation, national development and environmental protection.

**Methods:**

To strengthen health systems for delivery of context-specific, equity-focused reproductive, maternal, newborn and child health services (RMNCH), the Investment Case study was applied in the Asia-Pacific region. Staff of local and central government and non-government organisations analysed data indicative of health service delivery through a supply–demand oriented framework to identify constraints to RMNCH scale-up. Planners developed contextualised strategies and the projected coverage increases were modelled for estimates of marginal impact on maternal mortality and costs over a five year period.

**Results:**

In Indonesia, Philippines and Nepal the constraints behind incomplete coverage of family planning services included: weaknesses in commodities logistic management; geographical inaccessibility; limitations in health worker skills and numbers; legislation; and religious and cultural ideologies. Planned activities included: streamlining supply systems; establishment of Community Health Teams for integrated RMNCH services; local recruitment of staff and refresher training; task-shifting; and follow-up cards. Modelling showed varying marginal impact and costs for each setting with potential for significant reductions in the maternal mortality rate; up to 28% (25.1-30.7) over five years, costing up to a marginal USD 1.34 (1.32-1.35) per capita in the first year.

**Conclusion:**

Local health planners are in a prime position to devise feasible context-specific activities to overcome constraints and increase met need for family planning to accelerate progress towards MDG 5.

## Background

Unmet need for family planning is responsible for 7.4 million disability-adjusted life years [1] and 30% of the maternity-related disease burden [2]. By reducing high-risk pregnancies; those unwanted, in women above 40 or below 20 years of age, of high parity (above 5), with short birth intervals [3,4], family planning is estimated to reduce the risk of maternal death by up to 58% [5].

Family planning programs must serve to provide couples and sexually active women and men with correct information, quality services and timely access to affordable, safe, effective modern contraceptives with the provision of their method of choice. Yet, an estimated 200 million couples in developing countries presently state a desire to delay pregnancy or cease fertility but are not using modern contraception [6]. Beyond the access as a barrier, quality service is critical for effective and sustained use [7]. Inequity in those unserved is marked [8-10] in both utilisation of and ability to demand family planning [10,11]. In a sample of 41 developing countries the poorest wealth quintile was found to have twice the number of unwanted births than the richest (1.1 vs. 0.5) and endure less access to information and health workers [10]. In another analysis of 64 developing countries, inequitable unmet need for family planning was associated with area of residence, age and woman’s education, in addition to wealth [11].

Family planning is highly ranked for both impact and cost-effectiveness [5,12-17] and is a driver of progress towards other Millennium Development Goals (MDGs) [18-21]. Family planning lessens maternal mortality and morbidity through reduced total number of pregnancies, fewer unwanted pregnancies and associated unsafe abortions, and reduced numbers of high-risk pregnancies not receiving adequate obstetric care. The breadth of potential benefits include women’s empowerment, diminished poverty, and enabling of educational, social and economic participation [19]. Efficiency is particularly high in resource-constrained settings where reduced number of pregnancies partially offsets the required investment for emergency obstetric care (EmOC), the other pillar of maternal mortality reduction [13]. Satisfying the world’s unmet need for family planning could reduce maternal deaths by up to 29% [22] and in 2008 was estimated to cost only USD 1.20 per capita per year - totalling USD 6.7 billion annually of which USD 5.1 billion would be saved by reduced need for pregnancy and newborn care services [20]. Regardless, family planning remains under-resourced; in 2008 only 2.4% of the USD 10.6 billion of donor assistance for reproductive health was allocated to family planning [1] and actual dollar amounts have decreased since 2007 [20].

Family planning services in developing countries have not reached their potential as a result of several factors. Firstly, the reproductive health agenda has been vulnerable to wavering political commitment and cultural sensitivities. Secondly, competition for priority and divided advocacy has characterised the safe motherhood movement [19,23]. As noted recently by Diamond-Smith and Potts [24], the global commitment to scale-up skilled birth attendance and EmOC should not be at the expense of more immediate low-cost approaches such as family planning. Thirdly, the choice of maternal mortality ratio (MMR), instead of maternal mortality rate, as the key MDG-5 indicator shadows the positive role of family planning. Increasing contraceptive prevalence reduces the number of live births, the denominator of MMR, thus the absolute number of maternal lives saved is not reflected in the MMR. Finally, the supply and demand for family planning services are influenced by various constraints [25,26] unique to each setting which require assessment and context-specific responses [7,27].

To support governments in health system strengthening for scale-up of reproductive, maternal, newborn and child health (RMNCH) services, the Investment Case study was undertaken in Asia-Pacific region. The study facilitated local policymakers’ analysis of evidence of service delivery and development of equity-focused plans and budgets to accelerate progress on MDGs 4 and 5.

Specifically for family planning, this article presents the range of constraints, contextualised strategies and expected costs and mortality gains that emerged in three countries. Local planners demonstrated capacity to address context-specific constraints to family planning, affordably and within the health system, for impact on maternal mortality in the short term. This paper aims to show how health system strengthening through facilitation of context-specific, evidence-based planning supports efficient use of financial investment for accelerated scale-up of family planning.

## Methods

The Investment Case study combines quantitative data with qualitative problem-solving analysis. In a series of workshops, local, regional and central government health officials and service providers and staff of health sector non-government organisations convened to assess leading constraints to the delivery and uptake of essential RMNCH services. District- and region-specific data reflecting health status, intervention coverage, and service delivery was drawn on, through use of a framework originally developed by Tanahashi [28], for systematic analysis of supply, demand and quality factors influencing RMNCH.

**Figure 1:**
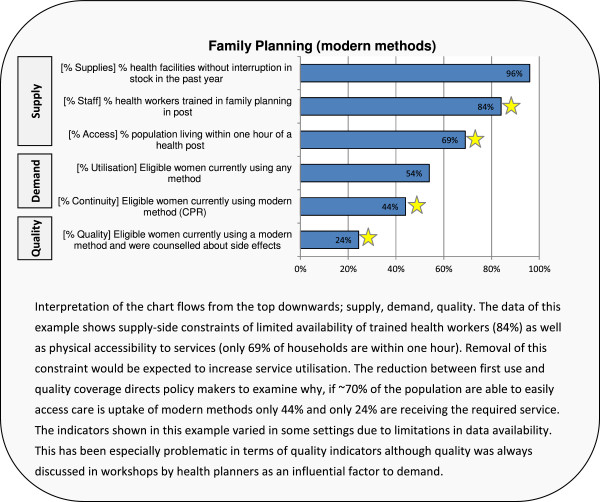
Framework for evidence-based analysis of supply and demand, example

An example of the framework of evidence for analysis is presented in Figure 1. Following this analysis, workshop participants developed feasible context-specific strategies to overcome constraints with a focus on disadvantaged and underserved population groups to accelerate scale-up. Planners aligned strategies with target increases in service coverage. Target setting was based on past demonstrated achievements in coverage in the country and local expertise as global literature shows no consistent patterns in the coverage levels attained by various strategies [29-31] and context and implementation are heavily influential [32,33]. To improve reliability, a ceiling was set for projected met need in line with the highest achievement of the region in which the study site was placed (based on Demographic Health Survey (DHS) data) to avoid excessively optimistic estimates by local planners. The ceilings were reached by planners of three sites in Indonesia (Merauke, Pontianak, and Tasikmalaya), two settings of the Philippines (Eastern and Northern Samar) and one site of Nepal (Terai). Further, a margin of ±10% of the proportion coverage increase was applied in the calculation of impact to accommodate uncertainty in both target setting and implementation. A process of validation of data, analysis, strategies and targets by in-country RMNCH experts and senior health officials was undertaken. Finally, the proposed activities and projected increases in coverage were modelled in a decision support tool [34] to estimate marginal impact on maternal, newborn and child mortality and costs over a five year period. This paper exclusively presents the results pertaining to family planning and maternal mortality, results of the full RMNCH package are presented elsewhere [35-37]. 

This work was carried out with ethical clearance from the University of Queensland, Medical Research Ethics Committee and the University of Queensland Behavioural & Social Sciences Ethical Review Committee, Australia. Written informed consent was obtained from participants for inclusion in the research and public availability of findings.

**Figure 2:**
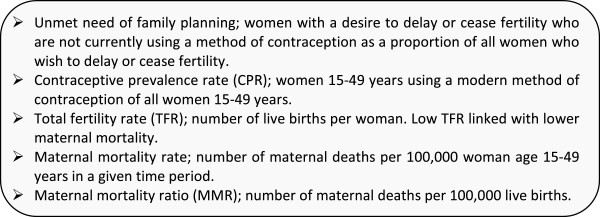
Key indicators in this analysis of family planning

The indicator of choice in this analysis; met need with a modern method of contraception, supports rights-based scale-up serving couples’ own fertility preferences [10,38] in comparison to contraceptive prevalence rate (CPR) targets which typically reflect the aim of policy makers rather than couples, although barriers do exist to a couple expressing need [10]. Definitions of need for family planning (shown in Figure 2) (the sum of unmet need and CPR, also termed total demand) used in the analysis were originally drawn from the country’s respective DHS [39-41] then have been adjusted applying the 2012 DHS revised algorithm for estimation of unmet need [42]. 

The estimated impact on maternal mortality draws on data regarding population of reproductive age women, national maternal mortality and aetiology, current and projected coverage of family planning moderated by contraceptive efficacy [43] (see Additional file 1: Appendix A).

Marginal cost estimates, summarised into categories of capital, human resources, drugs and medical supplies, and programmatic costs, incorporate all activities and health system costs for scale-up and changes in commodity consumption. Family planning scale-up frequently generates savings in the drugs and medical supplies category owing to fewer pregnancies and thereby less consumption of services, in particular the costs of antenatal and obstetric care, newborn and child health services.

This paper reports on the family planning components of the Investment Case study for: Indonesia (Merauke and Sikka districts, Pontianak and Tasikmalaya cities); Philippines (Pasay City, Eastern Samar and Northern Samar); and Nepal (clusters of districts of three geographic typologies; Mountains, Hills and Terai). In each country selection was based on two factors; greatest disadvantage and consultation with local governments.

### Data sources

In analysis of supply and demand constraints the study applied data from the following country-level sources: Indonesia - Susenas (National Basic Health Survey) 2007 [44], Riskesdas National Basic Health Survey 2008 [45], Provincial Health Information System [46], Indonesian NDHS 2007 [41]; Philippines – Philippines NDHS 2008 [40], Maternal Death Review [47], Pasay City Vital Statistics Division [48], Provincial Health Offices’ Annual Reports [49], Field Health Services Information System (FHSIS) 2007 [50]; and Nepal – Health Information Management System (HMIS), Department of Health Services Annual Report 2009–10 [51], Central Government Logistics Management Division, Nepal NDHS 2006 [39,52], National Living Standards Survey 2003–04 (NLSS) [53].

#### Findings

**Figure 3:**
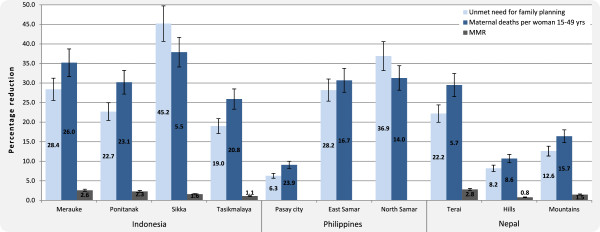
Projected outcomes for maternal health of three countries, by percentage reduction

**Figure 4:**
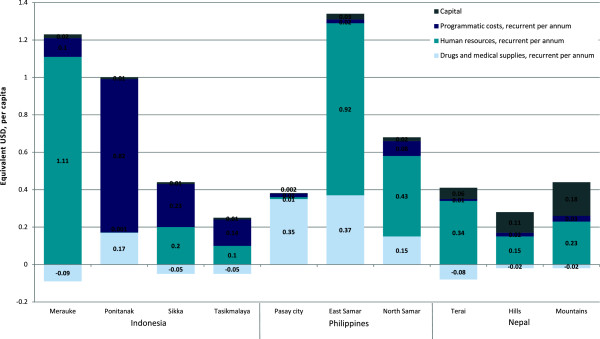
Marginal costs per capita, by category, of three countries, in equivalent USD

Disparities in maternal mortality, fertility and family planning coverage are evident both within and between countries, illustrated in Table 1. A series of themes emerged as constraints to family planning services: weakness in commodity supply systems; geographical inaccessibility; limited staff competence; human resources shortages; limited range of modern methods available; legislation; inadequate infrastructure; and cultural and religious ideologies. Strategies in response ranged from conventional to unique and innovative. Anticipated marginal impact was substantial with high feasibility; from 3.4% (3.1-3.6) to 27.9% (25.1-30.7) reduction in the maternal mortality rate costing a marginal USD 0.19 (0.20-0.19) to 1.34 (1.32-1.35) per capita in the first year. Full results are presented in Table 2, Figure 3 and Figure 4.

**Table 1:** Maternal mortality, fertility and family planning status, by country

**Indicator country**	**MMR (per 100,000 live births)**	**Total fertility rate (TFR)**	**CPR, modern method (married women aged 15–49)**	**Unmet need, modern method (married women aged 15–49)**	**Status of abortion services**
**Indonesia**	228 [41]	2.6 [41]	57.4 [42]	17.1 [42]	Illegal except if the pregnancy threatens the woman’s life, amongst other conditions [54].
**Merauke District**	--	3.4 ^Ϯ^[41]	42.8 [44]	31.7 ¤ [42,44]	
**Pontianak City**	--	2.8 ^Ϯ^[41]	46.7 [44]	27.8 ¤ [42,44]	
**Sikka District**	--	4.2 ^Ϯ^[41]	15.3 [44]	29.2 ¤ [42,44]	
**Tasikmalaya City**	--	2.6 ^Ϯ^[41]	49.5 [44]	25 ¤ [42,44]	
**Philippines**	162 [55]	3.3 ^Ϯ^[40]	34 [3]	38.7 [42]	Abortion is illegal. Abortion-related deaths not recorded as a cause of maternal death [56].
**Pasay City**	80 ^Ϯ^[48]	2.3 ^Ϯ^[40]	56 [50]	21.3 [40,42,50]	
**Eastern Samar**	160 ^Ϯ^[47]	4.3 ^Ϯ^[40]	37.5 [50]	33.0 [40,42,50]	
**Northern Samar**	160 ^Ϯ^[49]	4.3 ^Ϯ^[40]	15 [50]	55.5 [40,42,50]	
**Nepal**	281 [39]	3.1 [39]	44.2 [42]	28.4 [42]	Abortion legalised in 2002 and available in all districts [51].
**Terai**	--	3.1 [39]	47 [42,52]	16.5 [42,52]	
**Hills**	--	3.0 [39]	30.6 [42,52]	29.3 [42,52]	
**Mountain**	--	4.1 [39]	38.2 [42,52]	20.5 [42,52]	

-- Data not available.

^Ϯ^ Provincial / Regional level data.

¤ Indonesian Family Planning Bureau elected to use the National estimate of “need for family planning” for all regions for alignment with National policy.

**Table 2:** Summary of projected outcomes for three countries

**Region outcome**		**Indonesia**				**Philippines**			**Nepal**		
		**Merauke**	**Pontianak**	**Sikka**	**Tasikmalaya**	**Pasay city**	**East Samar**	**North Samar**	**Terai**	**Hills**	**Mountains**
**Unmet need (modern method)**											
baseline - target %		30.5 – 21.7	27.8 – 8.1	60.7 – 25.3	22.9 – 10.5	21.3 – 17.3	33 – 25.9	55.5 – 25.9	16.5 – 11.4	29.3 – 24.5	20.4 – 12.5
10% range on target		20.9 – 22.6	6.1 – 10.1	21.8 – 28.9	9.3 – 11.8	16.9 – 17.7	25.3 – 26.7	23 – 28.9	10.8 – 11.8	24 – 25	11.7 – 13.3
% reduction		8.7 %	19.7 %	35.3 %	12.4 %	4 %	7 %	29.6 %	5.1 %	4.7 %	7.9 %
**Maternal mortality rate**											
% reduction		9.4 %	21.6 %	27.9 %	13.9 %	3.4 %	6.9 %	14.1 %	7.8 %	5.2 %	10 %
10% uncertainty range		8.4 – 10.3	19.4 – 23.8	25.1 – 30.7	12.4 – 15.3	3.1 – 3.6	6.4 – 7.5	12.8 – 15.5	7.1 – 8.6	4.7 – 5.7	9 – 11
**MMR**											
% reduction		0.9 %	2.6 %	1.9%	1.0 %	Not calculated as abortion is not reported as a cause of maternal death.			1.7 %	0.8 %	2 %
10% uncertainty range		0.8 – 1.0	2.2 – 2.9	1.6– 2.1	0.7 – 1.1				1.6 – 1.9	0.7 – 0.8	1.8 – 2.3
**Annual health expenditure, per capita**											
Equivalent USD		National 77 (2010) [57]				National 77 (2010) [57]			National 29 (2010) [57]		
**Total marginal cost per capita**											
	Equivalent USD		1.14	1.0	0.39	0.19	0.39	1.34	0.68	0.32	0.26	0.42
10% uncertainty range		1.15 – 1.13	0.99 – 1.0	0.38 – 0.40	0.20 – 0.19	0.38 – 0.39	1.32 – 1.35	0.67 – 0.69	0.32 – 0.32	0.26 – 0.26	0.42 – 0.41	
**Marginal cost per capita, by category §** (equivalent USD)												
Capital		0.02	0.01	0.01	0.01	0.002	0.03	0.02	0.06	0.11	0.18	
Human resources, recurrent per annum		1.11	0.001	0.20	0.10	0.01	0.92	0.43	0.34	0.15	0.23	
Programmatic costs, recurrent per annum		0.10	0.82	0.23	0.14	0.02	0.02	0.08	0.01	0.02	0.03	
Drugs and medical supplies, recurrent per annum		−0.09	0.17	−0.05	−0.05	0.35	0.37	0.15	−0.08	−0.02	−0.02	

* Conversions: Indonesian Rupiah 2011 average USD 1 = 8500 IDR; Philippines Pesos 2010 average US 1 = 45.09 PHP; Nepalese Rupee not used.

§ Categories: Capital – upfront and pre-service training, infrastructure, other health system assets. Human resources – salaries, incentives, allowances, refresher training. Programmatic – intervention specific strategies and activities such as information, education and communication and behaviour change promotion, family planning program monitoring, supervision and evaluation. Drugs and medical supplies – commodities, buffer stock, medical supplies.

### Constraints to and strategies for the scale-up of family planning

#### Indonesia

The four sub-regions are characterised by low coverage of RMNCH services, inequity and disadvantage. Pontianak and Tasikmalaya are cities with high population densities but medium and extremely low fiscal capacities, respectively, and large private sectors which are minimally regulated. Sikka and Merauke are rural districts of limited infrastructure with very low and very high fiscal capacity, respectively, where communities rely almost solely on government village midwives for RMNCH services. Merauke’s distinct Papuan culture and extreme remoteness influence the very poor health outcomes.

### Constraints

Irregular **commodity supply systems** across the country, with particular severity in Sikka and Tasikmalaya, result from insufficient coordination between the District Health Department, Family Planning Programme Office and Family Planning Bureau. Critical **staff shortages** impede delivery of family planning in both Tasikmalaya and Sikka where only 47% and 52% [46] of midwife posts are filled. Sikka has been unable to attract and retain midwives as remote postings are characterised by hardship and a lack of ongoing support. Merauke is challenged by absences as staff prefer to reside near the city. **Limited competence of midwives** undermines demand and drives contraception discontinuation in Tasikmalaya, Pontianak and Sikka. Quality concerns extend to private sector midwives in the cities, notably in Pontianak where many foster repeat income by preferentially recommending short-term methods. Compounding this in Pontianak, temporary long-term methods are expensive and consumers have become suspicious following media publicity of complications which have been qualitatively reported as frequent. This is consistent with National data showing that, of all women discontinuing IUD and implant, 30.9% did so due to side effects and 26.6% due to health concerns [41]. **Community knowledge and understanding** of family planning is very low in Sikka where no promotional mechanisms are in place while in Merauke health promotion sessions are rare as Community Health Workers (CHWs) lack confidence performing this task. **Culture** encourages large family sizes in Sikka where the total fertility rate (TFR) of 4.2 dwarves the National average of 2.6 [41]. Fertility rates are also high in indigenous Papuan communities of Merauke in response to high infant mortality and a reluctance to accept ‘outsiders’ leaving many posted midwives underutilised.

### Strategies

Consistent **supply of commodities** for Sikka and Tasikmalaya is anticipated through annual meetings for the Family Planning Bureau and Health Office and streamlining in Sikka with peripheral midwives collecting commodities alongside routine report submission at central facilities. **Human resource shortages** in both Tasikmalaya and Sikka would be alleviated through recruitment of midwives for a 5.6% and 6.6% increase in staff numbers in each region. Planners scheduled annual training concerning short- and long-term contraceptives and management of complications for 7.3% and 23% of existing midwives in Tasikmalaya and Sikka each and 13% of public and private midwives in Pontianak. Quarterly supervision visits to all peripheral facilities by midwife coordinators (with supplementary remuneration) is expected to enhance **service quality**. Further targeting **midwives**’ **performance**; annual USD 5,000 for attendance allowances in Merauke; performance-based contracts with chiefs in all 160 villages of Sikka; and biannual Midwife Performance Competitions monitored by the Midwives Association (IBI) in Pontianak. Unique to each region, **community knowledge** was targeted through; annual interactive events in Tasikmalaya, integration of family planning with existing *Desa Siaga* (Village Awareness Program) by midwives in Sikka, and village forums with local CHWs trained for family planning promotion in Merauke. To generate **acceptance of long**-**term methods** in Pontianak city a ‘Complications Compensation Fund’ of USD 83,000 is to be annually dispersed, public patients receive free long-term methods, and private providers receive incentives for long-term methods.

### Projected outcomes

Planners projected unmet need to decrease from 30.5 to 21.7% (20.9-22.6) in Merauke, 27.8 to 8.1% (6.1-10.1) in Pontianak, 60.7 to 25.3% (21.8-28.9) in Sikka and 22.9 to 10.5% (9.3-11.8) in Tasikmalaya after five years and the maternal mortality rate to reduce by up to 9.4% (8.4-10.3), 21.6% (19.4-23.8), 27.9% (25.1-30.7) and 13.9% (12.4-15.3), respectively (see Figure 3).

Marginal expenditure, shown in Figure 4, was in line with the fiscal capacity of each region. Spending in Indonesia was largely consumed by annual recurrent programmatic costs with the exception of high human resource investment in Merauke. At most strategies require an additional 1.5% of Indonesia’s USD 77 per capita [57] annual health expenditure in 2010.

Strategies of Sikka were the most cost-effective, owing considerably to the very low baseline met need, reducing unmet need by 35.3% (31.8-38.9) for only USD 0.39 (0.38-0.40) per capita in the first year.

#### Philippines

With decentralisation in the Philippines, local government units (LGUs) are responsible for planning, budgeting and delivery of basic health services however are challenged by limited capacity.

The rural Northern and Eastern Samar provinces are characterised by high mortality, severe poverty and greatest disadvantage of the country. In contrast, RMNCH outcomes in urban Pasay City are above national average however inequity by wealth is significant.

### Constraints

The **supply of commodities** in all three regions is unreliable as local budgets have not incorporated commodities and management is weak since decentralisation. **Shortages and low competency of midwives** in Eastern and Northern Samar compromise service quality and hinder the functionality of Community Health Teams (CHTs) (coalitions of Barangay (Village) health workers, traditional birth attendants and supervising midwives tasked with health promotion, follow-up, and referrals). The present ratio of midwives to CHT of only one to four falls short of the National recommendation of one to one. In Northern Samar only 54% [49] of midwives have received the family planning refresher training. In Pasay City only 77% [48] of facilities have at least two providers trained in family planning and services of the large private sector are often considered low quality. The **legislation** imposed by several LGUs in Northern Samar limits family planning to ‘natural methods only’ and availability of modern contraceptives to only 32% [49] of the region’s facilities. Inadequate **community knowledge** constrains demand as CHTs lack leadership and do not effectively promote family planning, target eligible couples or follow-up. **Religious ideologies** linked with Catholicism, the predominant religion of the Philippines, renders family planning a sensitive topic and challenging to promote.

### Strategies

In all three regions **increases in availability**, **community awareness** and **quality** of family planning centred on **village midwives**; recruitment for increases of 33% for Northern and 53% for Eastern Samar of existing staff numbers to achieve one midwife for each Barangay and training at all 14 health centres in Pasay City to invigorate CHTs. The more effective CHTs, prioritising remote communities, are expected to extend the reach and integrate reproductive health with antenatal and child health services. With greater numbers, midwives are to update the ‘target client list’ and follow-up with family planning counselling as well as standard MNCH services. A more **consistent supply of commodities** should follow training for the concerned staff and addition of specific budget lines to avert shortfalls for the purchase of commodities. Limited availability of modern contraceptives is likely to remain in Northern Samar until LGUs reverse ‘natural methods only’ legislation. In Pasay City planners prioritised quality care with **capacity building** through annual refresher training on essential RMNCH protocols. This extends to private providers as compulsory training prior to issuance of the business licence to practice.

### Projected outcomes

Given five years, unmet need is expected to decrease from 21.3 to 17.3% (16.9-17.7) in Pasay city, 33 to 25.9% (25.3-26.7) in Eastern Samar and 55.5 to 25.9% (23–28.9) in Northern Samar. These achievements withstanding, up to 3.4% (3.1-3.6), 6.9% (6.4-7.5), and 14.1% (12.8-15.5) of the maternal mortality rate may be averted, respectively, as shown in Figure 3. Calculation of impact on MMR was precluded for the Philippines as abortion-related death data is not collected [56].

Strategies of Northern Samar has greatest efficiency; cost (per capita) 50% less than Eastern Samar for almost double the reduction in unmet need, and cost only 25% more than Pasay City for a 6-fold greater impact on unmet need. These differences are fundamentally influenced by baseline unmet need (shown in Table 2). Substantial spending for drugs and medical supplies resulted in the Philippines owing to the high cost of modern contraceptives and high use of less effective contraceptive methods. Marginal spending at most equates to only 1.7% of the annual USD 77 [57] spent per capita on health in 2010.

#### Nepal

Health planning and financing is a centralised process in Nepal although the Government seeks to build the capacity of district health officials for decentralised planning.

The Terai comprises substantial road networks, high population density and distinct cultural practices. In stark contrast, the Mountains’ population is sparse, roads are few, health facilities can take several days to reach and the TFR is highest in the country at 4.1 [39]. The challenges of the Hills lie between these two extremes.

### Constraints

For **medical equipment** facility managers traverse long distances with limited transport and seasonal inaccessibility to central distribution points and funds are not directed towards equipment purchasing. In the Terai, critical equipment is often missing while in the Hills there is a mismatch between facilities with family planning trained staff and those with supplies. **Geographical inaccessibility** is the leading barrier in the Hills and Mountains where only 72% and 63% [53] of families live within one hour of a health post, respectively. In the Hills and Mountains women are reluctant and often not permitted to travel alone and outreach is challenging for staff who also have security concerns. For women in the Hills, morning-only clinic opening hours conflict with farm work schedules. The **shortfall in auxiliary nurse midwives** (ANMs) is critical in the Terai where 36% [52] of ANMs posts are vacant rendering existing staff overworked and unmotivated. **Access to longterm contraceptive methods** is extremely limited, available at one or two facilities in each district, as few staff are able to administer long-term methods. **Poor quality of care** is common and staff report a lack of confidence in counselling and managing side-effects. Health supervisors in the Terai describe that high workload impedes frequent supervision and training of peripheral staff. **Religious and cultural ideologies** of Muslim communities in the Terai limit family planning uptake. **Inadequate infrastructure**, namely the absence of private rooms in most peripheral facilities, across the country precludes privacy and the administration of long-term methods.

### Strategies

All three regions planned **recruitment of ANMs** for peripheral facilities. In the Terai a 74% increase in ANMs is required to meet demands of the high population. In the Hills and Mountains, the modest 7% and 6.5% increases in ANMs, respectively, are expected to enable outreach sessions of greater number, duration and distance; a key strategy to **increasing accessibility** to remote communities. Allowances for safer accommodation in remote outreach sites was proposed for staff in the Hills and Mountains. **Infrastructure upgrades** with private examination rooms added to 10% of peripheral facilities and discretionary funds for equipment restock were included in budgets of all three regions. Uniquely in the Hills, planners intend to reschedule facility opening hours and review facilities to match those equipped with appropriately trained staff. Refresher training was proposed for all districts to **improve the quality** of counselling, management of complications and side effects by all ANMs, and up-skill selected ANMs for provision of long-term methods. To sustain **service improvements** senior ANMs would receive training and remuneration for regular supportive supervision visits to peripheral staff. Specifically in the Terai, task-shifting of family planning counselling to community health volunteers is expected to allay religion and culture-based concerns. Finally, a nationwide media campaign was proposed for generalised demand generation.

### Projected outcomes

Within five years, unmet need is anticipated to decrease from 16.5 to 11.4% (10.8-11.8) in the Terai, 29.3 to 24.5% (24–25) in the Hills, and 20.5 to 12.5% (11.7-13.3) in the Mountains with the maternal mortality rate reduced by 7.8% (7.1-8.6), 5.2% (4.7-5.7), 10% (9–11) in each region respectively. Spending was predominately categorised for annual recurrent human resources expenditure with modest capital investment associated with immediate, one-off training for staff. At most the strategies would demand USD 0.42 (0.42-0.41) per capita in the first year, only 1.4% of the USD 29 [57] annual health expenditure per capita.

## Discussion

The emergence of themes amongst the constraints to family planning scale-up afford a broad understanding of the issue however the real profit is in the unique details identified by local planners.

The literature documents broad areas of work for improving uptake of family planning: increased consumer knowledge; affordable services; consistent supply of contraceptives; removal of barriers to access [25]. Specific activities correlating with increased contraceptive prevalence and met need have included: media campaigns; interpersonal communications; cash transfers, voucher and savings schemes; social franchising; outreach; increased contraceptive options; and integration of services [7]. This evidence offers valuable guidance however the influence of context is increasingly seen as a key consideration for scale-up of health services [58] and contextual diversity as a complicating factor of the maternal health response [33].

Planners of all three countries included the rather conventional strategies of staff recruitment, training, supportive supervision, financial incentives and coordinated supply systems. In addition, and of great importance, planners responded to key rate limiting factors unique to each setting that potentially undermine the effectiveness of conventional actions. Particular context-specific strategies for more immediate increases in service uptake included user-friendly clinic hours, privacy in facilities, culturally and religiously-appropriate health messages and messengers, secure workplaces, inclusion of the private sector and village chiefs, targeted follow-up, increased contraceptive options, and task-shifting.

The Investment Case process holds value in the pursuit towards MDG-5 as the analysis diverts from an intervention perspective which naturally forces competition and priority setting between interventions, instead adopting a health system perspective. This encourages strategies that, while specific and targeted, support improved delivery of a range of interventions at multiple levels of the health system. Health system strengthening has shown merit in numerous settings [59] and may be essential for continued maternal mortality reductions [60].

**Figure 5:**
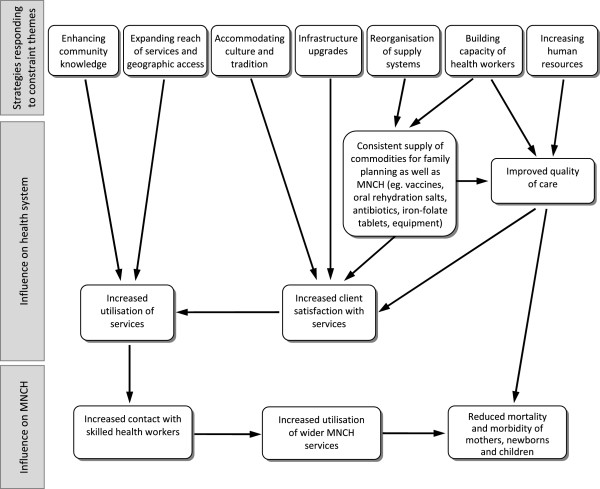
The interplay between strategies for scale-up of family planning and broader MNCH services

Even taking a basic analysis, in isolation from other MNCH interventions, the potential for family planning-oriented actions to contribute to increased coverage of other critical MNCH interventions is clear, illustrated in Figure 5, when a health system strengthening approach is adopted.

### Limitations

Two fundamental limitations to evidence-based planning are the availability and the quality of data. Data of service quality is particularly scarce as such indicators are not routinely collected in health information systems. The quality of reporting systems became an important discussion point amongst planners in the workshops.

The anticipated impact of strategies on maternal mortality is dependent on implementation achieving target coverage increases. Uncertainty is inherent to target setting although the study moderated for excessively optimistic or pessimistic coverage targets through three mechanisms: validation by relevant local experts and senior personnel; ceilings in line with the highest coverage achieved in region in which the study sites were placed in each country; and the +/− 10% uncertainty range. Estimates were considered feasible by RMNCH experts, particularly given the five year time frame, however programs are at the mercy of unpredictable local, national and global events.

The estimates of impact on maternal mortality presented are conservative as several factors influencing maternal survival could not be incorporated due to the current status of evidence.

Family planning for birth spacing (the interval between two consecutive live births) is linked with reduced risk of maternal death [61] however the evidence, particularly quantifying lives saved, has limitations – studies have frequently not been sufficiently powered to detect statistically significant changes in maternal mortality, or design has constrained the quality of findings [3].

Changes in access to abortion services were not included in the strategies and could not be accommodated in modelling due to limitations in data availability. As impact estimates of family planning are based only on the direct effect on unsafe abortions, results may underestimate the overall effect of the strategies on maternal mortality. Impact may be more obscured in Indonesia and Philippines compared to Nepal owing to the unavailability of legal, safe abortion services, often linked with higher incidence of unsafe abortion, in the former two countries however data shedding light on this factor is extremely limited.

Family planning and fertility data can be warped by a myriad of influences, with unmet need, abortion prevalence and abortion-related deaths holding particular relevance in the Investment Case. For instance, the TFR in Nepal has reduced from 3.1 [39] to 2.6 [62] between 2005–2010 however CPR has only increased from 48 [39] to 49.7 in that time - the gap possibly resulting from increased abortion and/or male absenteeism from outmigration. Underreporting of controversial abortion-related deaths would cause underestimation of impact in this study. These influential factors are key information gaps in reproductive health.

These limitations suggest that the estimated impact of family planning scale-up in this analysis is conservative and that incomplete evidence precludes us from observing the full benefits and cost-effectiveness of family planning services.

## Conclusion

A comprehensive maternal health approach delivering both family planning and maternal and newborn care (antenatal, emergency obstetric, newborn, and postpartum care) could prevent an estimated 70% of maternal deaths and 44% of newborn deaths [20]. In low resource settings, accessible, affordable, high quality EmOC is likely to be a gradual reality given the infrastructure and human resource requirements [58,63] and will be best supplemented with cost-effective interventions, such as family planning, that can reduce mortality in the short-term [5].

Overarching themes in responses to family planning constraints across the study sites may guide strategies in other settings; local staff recruitment, capacity building of relevant health workers with consistent supportive supervision and incentives, task-shifting, streamlining of supply systems, and culturally appropriate information, education and communication mechanisms.

In many countries national strategies cannot accommodate the diversity in health care provision and uptake. Understanding local factors may lead to more rapid scale-up of RMNCH services and progress towards MDGs. To this end, the inclusion of local-level staff in health system analysis and planning was invaluable and enabled the Investment Case study to unveil areas of opportunity where achievable, affordable, short-term interventions could overcome barriers to care. With increasing attention by international donors on scaling-up national family planning initiatives, it is important to recognise the potential impact of locally driven responses to system strengthening.

## Abbreviations

ANMs: Auxiliary nurse midwives; CHT: Community health teams; CHWs: Community health workers; CPR: Contraceptive prevalence rate; EmOC: Emergency obstetric care; LGUs: Local government units; MDG: Millennium development goals; MNCH: Maternal, newborn and child health; MMR: Maternal mortality ratio; TFP: Total fertility rate; USD: United states dollar.

## Competing interests

The authors declare that they have no competing interests.

## Authors' contributions

AB analysed and interpreted the data of the three countries and drafted the manuscript. AM coordinated the Investment Case study in Nepal and provided technical oversight regarding family planning and maternal mortality. EJS, as supervisor at University of Queensland as lead agency of the Investment Case study, coordinated across all countries and provided technical oversight of health system costing. ZD contributed to the study in Indonesia and the Philippines and analysis of data through the ‘decision-support tool’ in all countries for the IC study. ZD and AM conceived the separate family planning analysis from the wider Investment Case study. All authors contributed significantly to revision and approved the final manuscript.

## Supplementary Material

Additional file 1: Appendix A Family Planning Impact Calculations. Click here for file

## References

[B1] Tsui AO McDonald-Mosley R Burke AE Family planning and the burden of unintended pregnancies Epidemiol Rev 2010 32 1 152 174 10.1093/epirev/mxq012 20570955 PMC3115338

[B2] World Health Organization Department of Health Statistics and Informatics, Global health risks: mortality and burden of disease attributable to selected major risks 2009 Geneva: Switzerland

[B3] Conde-Agudelo A Rosas-Bermudez A Kafury-Goeta A Effects of birth spacing on maternal health: a systematic review Am J Obstet Gynecol 2007 196 4 297 308 10.1016/j.ajog.2006.05.055 17403398

[B4] Marston C Cleland J The effects of contraception on obstetric outcomes 2004 Geneva: Department of Reproductive Health and Research, World Health Organization

[B5] Prata N Sreenivas A Vahidnia F Potts M Saving maternal lives in resource-poor settings: facing reality Health Policy 2009 89 2 131 148 10.1016/j.healthpol.2008.05.007 18620778

[B6] World Health Organization Family planning: fact sheet No 351 2012 Geneva, Switzerland http://www.who.int/mediacentre/factsheets/fs351/en/index.html

[B7] Mwaikambo L Speizer I Schurmann A Morgan G Fikree G What works in family planning interventions: a systematic review Stud Fam Plann 2011 42 2 67 82 10.1111/j.1728-4465.2011.00267.x 21834409 PMC3761067

[B8] Gillespie D Contraceptive use and the poor: a matter of choice? PLoS Med 2007 4 2 e49 10.1371/journal.pmed.0040049 17284157 PMC1808089

[B9] Gakidou E Vayena E Use of modern contraception by the poor is falling behind PLoS Med 2007 4 2 e31 10.1371/journal.pmed.0040031 17284155 PMC1796626

[B10] Gillespie D Ahmed S Tsuia A Radloff S Unwanted fertility among the poor: an inequity? Bull World Health Organ 2007 85 2 100 107 10.2471/BLT.06.033829 17308730 PMC2636279

[B11] Ortayli N Malarcher S Equity analysis: identifying who benefits from family planning programs Stud Fam Plann 2010 41 2 101 108 10.1111/j.1728-4465.2010.00230.x 21466109

[B12] Kielmann AA Taylor CE Parker RL The Narangwal nutrition study: a summary review Am J Clin Nutr 1978 31 11 2040 2057 102180 10.1093/ajcn/31.11.2040

[B13] Goldie SJ Sweet S Carvalho N Natchu UCM Hu D Alternative strategies to reduce maternal mortality in India: a cost-effectiveness analysis PLoS Med 2010 7 4 e1000264 10.1371/journal.pmed.1000264 20421922 PMC2857650

[B14] Hu D Bertozzi SM Gakidou E Sweet S Goldie SJ The costs, benefits, and cost-effectiveness of interventions to reduce maternal morbidity and mortality in Mexico PLoS One 2007 2 8 e750 10.1371/journal.pone.0000750 17710149 PMC1939734

[B15] Jowett M Safe Motherhood interventions in low-income countries: an economic justification and evidence of cost effectiveness Health Policy 2000 53 3 201 228 10.1016/S0168-8510(00)00089-0 10996067

[B16] Prata N Sreenivas A Greig F Walsh J Potts M Setting priorities for safe motherhood interventions in resource-scarce settings Health Policy 2010 94 1 1 13 10.1016/j.healthpol.2009.08.012 19773090

[B17] Walsh JA Measham AR Feifer CN Gertler PJ The impact of maternal health improvement on perinatal survival: cost-effective alternatives Int J Health Plann Manage 1994 9 2 131 149 10.1002/hpm.4740090203 10137136

[B18] World Health Organization Repositioning family planning: guidelines for advocacy action 2008 Washington DC: WHO, USAID

[B19] Cleland J Bernstein S Ezeh A Faundes A Glasier A Innis J Family planning: the unfinished agenda Lancet 2006 368 9549 1810 1827 10.1016/S0140-6736(06)69480-4 17113431

[B20] Singh S Darroch J Ashford L Vlassoff M Adding it up: The costs and benefits of investing in family planning and maternal and newborn health 2009 New York: Guttmacher Institute and United Nations Population Fund

[B21] Canning D Schultz T The economic consequences of reproductive health and family planning Lancet 2012 380 9837 165 171 10.1016/S0140-6736(12)60827-7 22784535

[B22] Ahmed S Li Q Liu L Tsui A Maternal deaths averted by contraceptive use: an analysis of 172 countries Lancet 2012 380 9837 111 125 10.1016/S0140-6736(12)60478-4 22784531

[B23] Shiffman J Smith S Generation of political priority for global health initiatives: a framework and case study of maternal mortality Lancet 2007 370 9595 1370 1379 10.1016/S0140-6736(07)61579-7 17933652

[B24] Diamond-Smith N Potts M A woman cannot die from a pregnancy she does not have Int Perspect Sex Reprod Health 2011 37 3 155 10.1363/3715511 21988792

[B25] Prata N Making family planning accessible in resource-poor settings Phil Trans R Soc 2009 364 1532 3093 3099 10.1098/rstb.2009.0172 PMC2781837 19770158

[B26] Culwell K Vekemans M de Silva U Hurwitz M Crane B Critical gaps in universal access to reproductive health: contraception and prevention of unsafe abortion Int J Gynaecol Obstet 2010 110 Supple S13 S16 20451196 10.1016/j.ijgo.2010.04.003

[B27] Cottingham J Germain A Hunt P Use of human rights to meet the unmet need for family planning Lancet 2012 380 9837 172 180 10.1016/S0140-6736(12)60732-6 22784536

[B28] Tanahashi T Health service coverage and its evaluation Bull World Health Organ 1978 56 2 295 303 96953 PMC2395571

[B29] Dudley L Garner P Strategies for integrating primary health services in middle- and low-income countries at the point of delivery Cochrane Database System Rev 2011 6 7 CD003318 16625576 10.1002/14651858.CD003318.pub2

[B30] Wallace A Dietz V Cairns K Integration of immunization services with other health interventions in the developing world: what works and why? Systematic literature review Trop Med Int Health 2009 14 1 11 19 10.1111/j.1365-3156.2008.02196.x 19017307

[B31] Lopez L Hiller J Grimes D Chen M Education for contraceptive use by women after childbirth Cochrane Database Syst Rev 2012 8 CD001863 22895923 10.1002/14651858.CD001863.pub3

[B32] Hollingworth S Hertz D Malik ASF Dettrick Z Jimenez Soto E Maternal child health strategies database: evidence for strategies to improve health Health Res Policy Syst 2012 Under review, 1st October 2012 http://www.uq.edu.au/investmentcase/tools-resources

[B33] Campbell OMR Graham WJ Lancet Maternal Survival Series steering group Strategies for reducing maternal mortality: getting on with what works Lancet 2006 368 9543 1284 1299 10.1016/S0140-6736(06)69381-1 17027735

[B34] Jimenez-Soto E La Vincente S Clark A Firth S Morgan A Dettrick Z Dayal P Aldaba B Varghese B Trisnantoro L Prasai Y Investment Case team for India, Indonesia, Nepal, Papua New Guinea, and the Philippines Developing and costing local strategies to improve maternal and child health: the Investment Case framework PLoS Med 2012 9 8 e1001282 10.1371/journal.pmed.1001282 22879817 PMC3413720

[B35] Trisnantoro L Wdiati Y Kurniawan F Harbianto D Jimenez-Soto E Dettrick Z Firth S UNICEF Indonesia Indonesia: developing an investment case for financing equitable progress towards MDGs 4 and 5 in the Asia Pacific region (scale-up report) 2011 Brisbane, Australia: Gadjah Mada University, University of Queensland, UNICEF Indonesia

[B36] Aldaba B La Vincente S Kraft A Jimenez-Soto E Dettrick Z Firth S Philippines Investment Case team Philippines: developing an investment case for financing equitable progress towards MDGs 4 and 5 in the Asia Pacific region (scale-up report) 2011 Brisbane, Australia: UPecon Inc., Centre for International Child Health at Murdoch Children's Research Institute, University of Queensland, UNICEF Philippines

[B37] Morgan A Prasai Y Jimenez-Soto E Dettrick Z Firth S Nepal: developing an investment case for financing equitable progress towards MDGs 4 and 5 in the Asia Pacific region (scale-up report) 2011 Brisbane, Australia: New ERA, Nossal Institute for Global Health, University of Queensland, UNICEF Nepal

[B38] Dixon-Mueller R Germain A Fertility regulation and reproductive health in the Millennium Development Goals: The search for a perfect indicator Am J Public Health 2007 97 1 45 51 10.2105/AJPH.2005.068056 16571693 PMC1716248

[B39] Ministry of Health and Population Nepal New ERA Macro International Inc Nepal demographic and health survey 2006 2007 Kathmandu, Nepal: Ministry of Health and Population

[B40] National Statistics Office Philippines ICF Macro Philippines national demographic and health survey 2008 2008 Calverton, Maryland, United States: National Statistics Office and ICF Macro

[B41] Statistics Indonesia (Badan Pusat Statistik-BPS) Macro International Inc Indonesia demographic and health survey 2007 2008 Calverton, Maryland, USA: BPS and Macro International

[B42] Bradley S Croft T Fishel J Weston C Revising unmet need for family planning 2012 Maryland USA: ICF International

[B43] Trussell J Hatcher R, Trussell J, Nelson A, Cates W, Stewart F, Kowal D Contraceptive efficacy Contraceptive technology 2007 19 New York: Ardent Media

[B44] Central Board of Statistics (BPS) of Indonesia Indonesian susenas (national socioeconomic survey) 2007 2007 Jakarta: Central Bureau of Statistics

[B45] National Institute of Health Research and Development Riskesdas (national basic health survey) 2008 2008 Jakarta: Ministry of Health, Republic of Indonesia

[B46] Centre for Data and Information Health information system Indonesia: Annual report 2009 Jakarta, Indonesia: Ministry of Health Indonesia

[B47] East Samar Provincial Health Office Maternal death review 2009 Manila, Philippines: Department of Health

[B48] Pasay City Health Office Pasay city vital statistics 2008 Manila, Philippines: Department of Health

[B49] North Samar Provincial Health Office Annual report 2008 Manila, Philippines: Department of Health

[B50] Tayag E Roque V De Los Reyes V Hernaez J Cantero J Toledo K Bautista F Lameda L Field health services information system (FHSIS) 2008 2010 Manila, Philippines: Division of Public Health Surveillance and Informatics, National Epidemiology Center

[B51] Department of Health Services, Nepal Annual report 2066/67 (2009/10) 2011 Kathmandu, Nepal: Ministry of Health and Population

[B52] Ministry of Health and Population Nepal New ERA Macro International Inc Nepal demographic and health survey 2006 - disaggregated data of cluster of districts 2008 Kathmandu, Nepal: Ministry of Health and Population

[B53] Central Bureau of Statistics Nepal National living standards survey 2003–04 (NLSS) - volume one 2004 Kathmandu, Nepal: CBS, National Planning Commission, Government of Nepal

[B54] Sedgh G Ball H In brief; abortion in indonesia, No 2 2008 New York: Guttmacher Institute 19035004

[B55] Philippines National Statistics Office Press release - Philippines family planning survey 2006: Preliminary results 2007 Manila: National Statistics Office, Republic of the Philippines

[B56] Singh S Ball H Hussain R Nadeau J Unintended pregnancy and induced abortion in the Philippines: causes and consequences 2006 New York: Guttmacher Institute

[B57] World Health Organization Global health expenditure database Accessed July 2012 (2010 data):http://apps.who.int/nha/database/PreDataExplorer.aspx?d=1

[B58] Mangham L Hanson K Scaling up in international health: what are the key issues? Health Policy Plan 2010 25 2 85 96 10.1093/heapol/czp066 20071454

[B59] Leatherman S Ferris T Berwick D Omaswa F Crisp N The role of quality improvement in strengthening health systems in developing countries Int J Qual Health Care 2010 22 4 237 243 10.1093/intqhc/mzq028 20543209

[B60] Freedman L Health system strengthening: new potential for public health and human rights collaboration Reprod Health Matters 2007 15 30 219 220 10.1016/S0968-8080(07)30323-6 17938089

[B61] Conde-Agudelo A Belizian J Maternal mortality associated with inter-pregnancy interval: cross sectional study BMJ 2000 321 1255 1259 10.1136/bmj.321.7271.1255 11082085 PMC27528

[B62] Ministry of Health and Population Nepal New ERA Macro International Inc Nepal demographic and health survey 2011 2011 Kathmandu, Nepal: Ministry of Health and Population

[B63] Kerber K de Graft-Johnson J Bhutta Z Okong P Starrs A Lawn J Continuum of care for maternal, newborn, and child health: from slogan to service delivery Lancet 2007 370 9595 1358 1369 10.1016/S0140-6736(07)61578-5 17933651

