# Supplementary material for: Context-specific, evidence-based planning for scale-up of family planning services to increase progress to MDG 5: health systems research
Source: Reprod Health. 2012 Nov 12;9:27. doi: 10.1186/1742-4755-9-27 (PMC3563623; doi:10.1186/1742-4755-9-27)
Supplement: Additional file 1: Appendix A — Family Planning Impact Calculations. [file 1742-4755-9-27-S1.pdf]

Appendix A- Family Planning Impact Calculations

The following section describes the way in which the impact of increased family planning coverage on mortality and number of pregnancies is calculated. Figure 1 presents a brief overview of the steps, with the sections following detailing the specific formulae used.

Figure 1: Overview of Family Planning Calculations

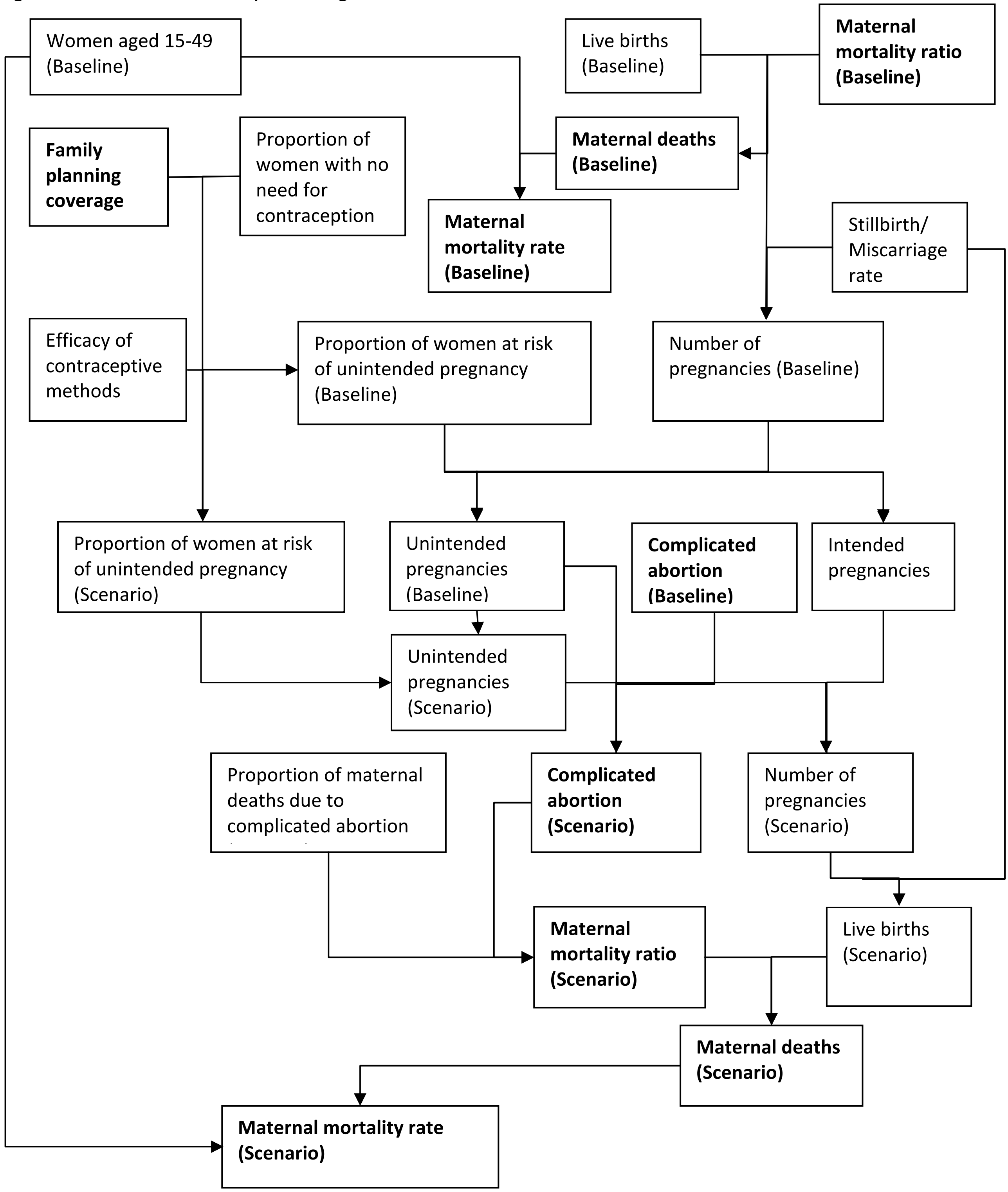

Byrne A, Morgan A, Jimenez Soto E, Dettrick Z: **Context-specific, evidence-based planning for scale-up of family planning services to increase progress to MDG 5: health systems research**, *Reproductive Health* 2012, **9**(27).

Calculating efficacy of family planning (both in baseline and scenario):

The efficacy of family planning in preventing pregnancy is calculated based on individual method efficacies[1] and the proportion of family planning users using each method. This is done so that the total number of pregnancies can be adjusted to reflect changes in the mix of contraceptive methods used – if more effective methods are used then the number of pregnancies due to contraceptive failure will decrease. The total efficacy of the method mix is calculated using the following formula:

$$E_{Total} = (E_{m1} \times M_{m1}) + (E_{m2} \times M_{m2}) + (E_{m3} \times M_{m3}) \dots$$

Where
E_{Total} = Total efficacy of modern methods in preventing pregnancy
E_m = Efficacy of individual method in preventing pregnancy
M_m = Proportion of family planning users using individual method

Calculating proportion of women at risk of having an unintended pregnancy (both in baseline and scenario):

In order to estimate the change in the number of pregnancies as a result of greater family planning coverage, the proportion of women in the population who are at risk of having an unintended pregnancy is calculated. This is done by excluding all women who are effectively covered by a contraceptive method from the pool of women with a need for family planning, using the following formula:

$$UnR = N \times (1 - (FP \times E_{Total})$$

Where:
UnR = Proportion of women 15-49 at risk of having an unintended pregnancy
N = Proportion of women 15-49 with need for family planning
FP = Total coverage of modern family planning methods (either baseline or scenario)
E_{Total} = Total efficacy of modern methods in preventing pregnancy (either baseline or scenario)

Note: The same formula is used to calculate both $UnR_{Baseline}$ and $UnR_{Scenario}$ by using either $FP_{Baseline}$ or $FP_{Scenario}$. If the mix of family planning methods used is changed, E_{Total} may also be recalculated for the scenario.

[1] Trussell J: **Contraceptive efficacy.** In *Contraceptive Technology.* Nineteenth Revised edition. Edited by Hatcher R, Trussell J, Nelson A, Cates W, Stewart F, Kowal D. New York: Ardent Media; 2007

Calculating the number of pregnant women in a scenario:

The total number of pregnancies in the scenario will change as a result changes in the proportion of the population at risk of pregnancy. Firstly, the number of intended and unintended pregnancies at baseline is estimated, using the following formulas:

$$Pr_{Baseline} = \frac{LB_{Baseline}}{(SB + \ MMR_{Baseline})}$$

$$PrI_{Baseline} = Pr_{Baseline} \times \frac{InR_{Baseline}}{InR_{Baseline} + UnR_{Baseline}}$$

$$PrU_{Baseline} = Pr_{Baseline} - \ PrI_{Baseline}$$

Where:
$Pr_{Baseline}$ = Number of pregnant women in cohort at baseline
$PrI_{Baseline}$ = Number of pregnant women with intended pregnancies at baseline
$PrU_{Baseline}$ = Number of pregnant women with unintended pregnancies at baseline
$InR_{Baseline}$ = Proportion of women 15-49 intending to become pregnant soon
$UnR_{Baseline}$ = Proportion of women 15-49 at risk of having an unintended pregnancy at baseline
$LB_{Baseline}$ = number of live births in baseline
SB = still birth rate (as a proportion of live births)
$MMR_{Baseline}$ = maternal mortality ratio in baseline

Next, the ratio of unwanted pregnancies to the proportion of women at risk of having an unwanted pregnancy is calculated. This is then used to estimate the total number of unwanted pregnancies in the scenario based on changes in the proportion of women at risk. The following formulas were used:

$$R = \frac{PrU_{Baseline}}{UnR_{Baseline} \ \times W}$$

$$PrU_{Scenario} = Pr_{Baseline} \ \times UnR_{Scenario} \times R$$

$$Pr_{Scenario} = PrI_{Baseline} + \ PrU_{scenario}$$

Where:
$Pr_{Baseline}$ = Number of pregnant women at baseline
$Pr_{Scenario}$ = Number of pregnant women in scenario
$PrI_{Baseline}$= Number of pregnant women with intended pregnancies at baseline
$PrU_{Baseline}$ = Number of pregnant women with unintended pregnancies at baseline

$PrU_{Scenario}$ = Number of pregnant women with unintended pregnancies in scenario
$UnR_{Baseline}$ = Proportion of women 15-49 at risk of having an unintended pregnancy at baseline
$U_{Scenario}$ = Proportion of women 15-49 at risk of having an unintended pregnancy in scenario
W = Number of women 15-49
R = Number of unwanted pregnancies per woman at risk of unintended pregnancy

Note: There is an assumption that the number of unwanted pregnancies per woman at risk of having an unwanted pregnancy will be stable between baseline and scenario. Without targeted efforts to change sexual habits or abortion-seeking behaviours (not included in any of our scenarios) it is unlikely that this ratio would change significantly.

Calculating effect of increased family planning coverage on complicated abortion:

The overall incidence of complicated abortion in pregnancy is adjusted so that it represents the risk in those with unintended pregnancies, rather than the risk in the entire population. This is done based on the assumption that unwanted pregnancies prevented by family planning are the pregnancies that will result in complicated abortion. The following formula is used:

$$A_{Unintended} = A_{Baseline} \times (\frac{Pr_{Baseline}}{PrU_{Baseline}})$$

Where:
$A_{Baseline}$ = proportion of all pregnancies resulting in complicated abortion in baseline
$A_{Unintended}$ = proportion of unintended pregnancies resulting in complicated abortion
$PrU_{Baseline}$ = Number of pregnant women with unintended pregnancies at baseline
$Pr_{Baseline}$ = Number of pregnant women in cohort at baseline

This risk is then applied to the proportion of women with unwanted pregnancies in the scenario and readjusted to reflect the new incidence in the total population using the following formula:

$$A_{Scenario} = A_{Unintended} \times \left(\frac{PrW_{Scenario}}{Pr_{Scenario}}\right)$$

Where:
$A_{Scenario}$ = proportion of pregnancies resulting in complicated abortion in scenario
$A_{Unintended}$ = proportion of unintended pregnancies resulting in complicated abortion
$PrU_{Baseline}$ = Number of pregnant women with unintended pregnancies at baseline
$PW_{Baseline}$ = Number of pregnant women in cohort at baseline

Note: There is an assumption that there will be no complicated abortions in women with wanted pregnancies, and that the risk of complicated abortion in women with unwanted pregnancies will also remain the same between baseline and scenario

Calculating impact of increased family planning coverage on maternal mortality ratio

As discussed in the main text, only the direct impact of family planning in preventing complicated abortions is used to modify the MMR. The effect of family planning on the total number of maternal deaths is incorporated as part of the calculations regarding the total number of pregnancies noted above. The following calculation is used to calculate the reduction in overall MMR as a result of decreased abortion incidence:

$$R_{Abortion} = \frac{A_{Baseline} - A_{Scenario}}{A_{Baseline}}$$

$$MMR_{Scenario} = MMR_{Baseline} \times (1 - (D_{Abortion} \times R_{Abortion}))$$

Where:

$MMR_{Scenario}$ = maternal mortality ratio in scenario
$MMR_{Baseline}$ = maternal mortality ratio in baseline
$A_{Scenario}$ = proportion of pregnancies resulting in complicated abortion in scenario
$A_{Baseline}$ = proportion of pregnancies resulting in complicated abortion in baseline
$R_{Abortion}$ = proportional reduction in occurrence of complicated abortion
$D_{Abortion}$ = proportion of maternal deaths as a result of complicated abortion

Note: There is an assumption that in the absence of appropriate medical care every complicated abortion will result in maternal death. As coverage of post-abortion care is included as a separate intervention, and was not modified in any of the scenarios, this makes the assumption that a change in mortality due solely to a change in complicated abortion incidence a reasonable one.

Calculating total impact of increased family planning coverage on maternal mortality rate

Family planning's effect on the maternal mortality rate, as opposed to the maternal mortality ratio, is calculated using both the direct impact on abortion deaths mentioned above and the indirect effects of decreasing the number of pregnancies. The measures are combined in the following way:

$$MD_{Baseline} = MMR_{Baseline} \times LB_{Baseline}$$

$$LB_{Scenario} = Pr_{Scenario} - (Pr_{Scenario} \times (SB + MMR_{Scenario}))$$

$$MD_{Scenario} = MMR_{Scenario} \times LB_{Scenario}$$

$$Maternal\ Mortality\ Rate = \frac{MD_{Scenario}}{W}$$

Where:

$MD_{Scenario}$ = number of maternal deaths in scenario
$MD_{Baseline}$ = number of maternal deaths in baseline
$MMR_{Scenario}$ = maternal mortality ratio in scenario
$MMR_{Baseline}$ = maternal mortality ratio in baseline
$LB_{Scenario}$ = number of live births in scenario
$LB_{Baseline}$ = number of live births in baseline
$Pr_{Scenario}$ = number of pregnant women in scenario
SB = still birth rate (as a proportion of live births)
W = Number of women 15-49

Note: These calculations assume that pregnancies that result in a maternal death will not result in a live birth. This is somewhat problematic as maternal deaths range from those occurring in pregnancy (where the likelihood of child survival is very low) to those occurring 42 days post-partum (where the likelihood of child survival is reasonable). As the proportion of deaths occurring at each point varies, and the probability of infant survival is highly context specific, it was not feasible to incorporate these factors into the calculation at this time.
